# Geometrical Determinants of Neuronal Actin Waves

**DOI:** 10.3389/fncel.2017.00086

**Published:** 2017-03-29

**Authors:** Caterina Tomba, Céline Braïni, Ghislain Bugnicourt, Floriane Cohen, Benjamin M. Friedrich, Nir S. Gov, Catherine Villard

**Affiliations:** ^1^Université Grenoble Alpes, Centre National de la Recherche Scientifique (CNRS), Institut NéelGrenoble, France; ^2^Université Grenoble Alpes, Centre National de la Recherche Scientifique (CNRS), Laboratoire des Technologies de la Microélectronique, CEA-LETIGrenoble, France; ^3^Laboratoire PhysicoChimie Curie, Institut Curie, Pierre-Gilles de Gennes Institute for Microfluidics, CNRS, PSL Research UniversityParis, France; ^4^Biological Algorithms Group, Center for Advancing Electronics Dresden, Technische Universität DresdenDresden, Germany; ^5^Department of Chemical Physics, Weizmann Institute of ScienceRehovot, Israel

**Keywords:** axon, polarization, actin wave, micropatterns, modeling

## Abstract

Hippocampal neurons produce in their early stages of growth propagative, actin-rich dynamical structures called actin waves. The directional motion of actin waves from the soma to the tip of neuronal extensions has been associated with net forward growth, and ultimately with the specification of neurites into axon and dendrites. Here, geometrical cues are used to control actin wave dynamics by constraining neurons on adhesive stripes of various widths. A key observable, the average time between the production of consecutive actin waves, or mean inter-wave interval (IWI), was identified. It scales with the neurite width, and more precisely with the width of the proximal segment close to the soma. In addition, the IWI is independent of the total number of neurites. These two results suggest a mechanistic model of actin wave production, by which the material conveyed by actin waves is assembled in the soma until it reaches the threshold leading to the initiation and propagation of a new actin wave. Based on these observations, we formulate a predictive theoretical description of actin wave-driven neuronal growth and polarization, which consistently accounts for different sets of experiments.

## Introduction

Growing neuronal branches (neurites) regularly exhibit propagative actin-based membrane deformations, or actin waves, which travel from the cellular body (soma) to the neurite tip. Actin waves are physiological events generic for a large set of mammalian neurons, e.g., of hippocampal or cortical origin. They have been observed both in dissociated neurons in culture and within brain slices (Katsuno et al., 2015; Flynn et al., 2009). The role of these directional structures in neuronal growth and polarization was already underlined in the first descriptions of this phenomenon in the literature, about two decades ago (Ruthel and Banker, 1998). Actin waves are associated with outbursts of neurite growth following the possible reactivation of the growth cone, and thereby contribute to the fast elongation of the nascent axon. Interestingly, these outbursts result from two successive events occurring at the tip: the tip first retracts as soon as the actin wave leaves the soma, and then grows forward when this actin wave merges with the growth cone. This suggests that actin waves, as already proposed in the seminal work of Ruthel and Banker (Ruthel and Banker, 1999), might create tension and exert pulling forces along neurites, which are important players implied in neurite growth and axonal specification (Lamoureux et al., 2002; Franze and Guck, 2010). As compared to non-neuronal actin waves [e.g., actin ruffles (Goicoechea et al., 2006), circular waves (Bernitt et al., 2015) and planar waves (Sun et al., 2015)], neuronal actin waves have remained poorly studied for many years, and the wave generation mechanism still remains elusive. Several in-depth studies published in recent years have however provided new insights into the role and characteristics of these propagative structures. First, actin waves seem to propagate using directional actin treadmilling that generates mechanical force (Katsuno et al., 2015). Actin polymerization and depolymerization is accompanied by the presence of several actin-associated proteins, such as Arp3, cofilin, or shootin within the actin wave (Flynn et al., 2009; Toriyama et al., 2006; Tilve et al., 2015). Interestingly, the wave of actin polymerization directs microtubule-based transport by triggering an upstream wave of microtubule polymerization mediated by a transient widening of the neurite shaft (Winans et al., 2016). This picture is in agreement with the presence of microtubule-associated proteins like doublecortin within actin waves (Tint et al., 2009). Finally, it should be noted that fin-like propagative structures similar to neuronal actin waves have been observed in 3T3 cells grown on nanofibers, conferring to these non-neuronal cells neurite-like protrusions (Guetta-Terrier et al., 2015).

In a previous study, we have shown that the control of the neurite width using micropatterns of adhesion yielded a fine tuning of both the length of neurites and the localization of axonal specification (Tomba et al., 2014). To consider the role of actin waves in neuronal growth, in the present work we have used predetermined neuronal morphologies to get new insight into the geometrical determinants of the actin wave dynamics, including temporal periodicity and induced neurite growth. This allowed us to develop a predictive model of neurite length and axonal polarization using the neurite width-dependent characteristics of actin waves. We further confronted this model to various sets of neuronal morphologies and found overall good agreement between both.

## Methods

### Neuron culture, labeling, and imaging

Embryonic day E18 mice hippocampi of Oncins France 1 or C57BL/6J mouse (Charles River) were dissected. We used both strains depending on the availability of animals at the time of culture (we did not encounter any statistically significant differences in actin wave dynamics between the two strains, as repeatedly checked on different patterns before merging the data). These hippocampi were then dissociated in MEM medium supplemented with 10% horse serum, 1% L-Glutamine, 1% Sodium pyruvate, and 0.05% Peni-streptomycine (Invitrogen). Neurons were plated on Poly-L-Lysine (Sigma, P2636) coated coverslips and kept first (for 3 h) in the dissociation medium and then in the maintenance medium of Neurobasal supplemented with 2% B27, 1% L-Glutamine, and 0.05% Peni-streptomycine (Invitrogen). Neurons were fixed in 4% paraformaldehyde and immunostained with standard techniques, after a permeabilization step of 30 min in PBS supplemented with 2% BSA and 0.25% Triton. The following antibodies and respective secondary antibodies were used in the indicated dilutions: for microtubules, rat anti-tubulin antibody (clone YL1/2, 1:500) and Alexa488 coupled (Invitrogen, 1:250), for axons, Tau (clone Tau-1, Millipore, 1:500), and CY3 coupled (Invitrogen, 1:250), for DNA, Hoechst (Invitrogen, 1:1000).

Isolated fixed neurons were analyzed with an upright Olympus BX51 microscope using a x20 dry objective (LMPLFL20x from Olympus, NA 0.4) and a F-View camera. Images of living neurons were acquired using three different inverted microscopes equipped with x20 dry, phase contrast objectives (NA 0.4): Olympus IX71 (Hamamatsu ORCA-ER camera), Zeiss Axiovert 200M (AxioCam camera), and Leica microscope DMi8 (Hamamatsu ORCA Flash 4.0 V2 camera). All were equipped with a heated work plate, a humidifier, a CO_2_ delivery system and a motorized stage to allow multi-position and multi-condition acquisitions. Images were acquired every 2–4 min. Only propagative growth cone-like structures were counted as actin waves (see Fig. S1).

The study was carried out in accordance with European Community guidelines on the care and use of laboratory animals: 86/609/EEC. The research purpose and the protocol are described in the Ethical Annex of ERCadg project CellO, which was approved and is regularly reviewed by the ERCEA. Institut Curie animal facility has received license #C75-05-18, 24/04/2012, reporting to Comité d'Ethique en matiére d'expérimentation animale Paris Centre et Sud (National registration number: #59).

### Statistical tests

**Figure 1:**
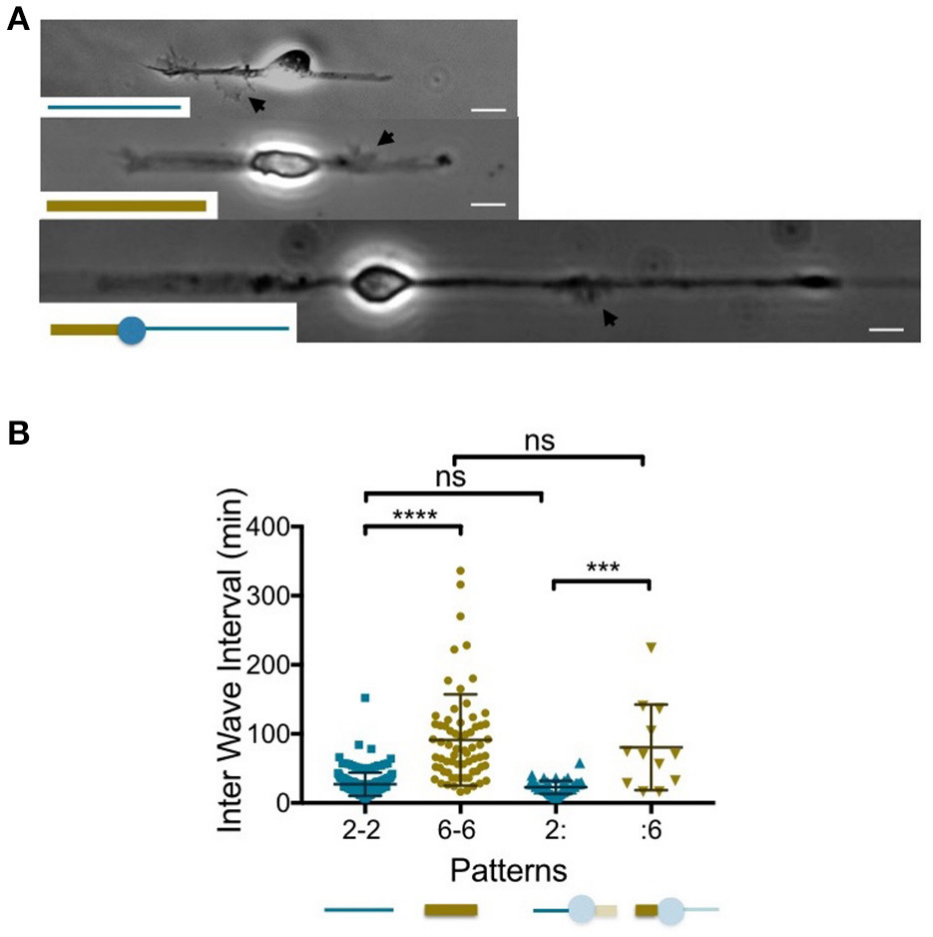
**Temporal patterns of waves according to the neurite width. (A)**. Images of 1 day *in vitro* neurons grown on 2 μm (top) and 6 μm (middle) wide adhesive stripes and a combination of both (bottom). Black arrows point to actin waves. Adhesive patterns are represented in the insets. Scale bars:10 μm. **(B)** Distribution of the time intervals between two successive actin waves computed at the neurite level (Inter Wave Intervals: IWI) for the three different neuron geometries shown in **(A)**. The corresponding patterns are represented below the graph using the same color code as the one chosen to plot the IWI distributions (blue and gold for the 2 μm and 6 μm wide stripes, respectively). The neurites of interest for the 2:6 pattern appear opaquely. Mean values and *SD*: 27.1 ± 16.8 min (2-2, *n* = 173, 4 cells), 91.1 ± 65.9 min (6-6, *n* = 68, 6 cells), 22.7 ± 9.5 min (2: *n* = 45, 4 cells), 80.5 ± 61.8 min (:6, *n* = 12, 4 cells). ns: *p* > 0.5, ^***^p < 0.004, ^****^p < 0.0001.

**Figure 2:**
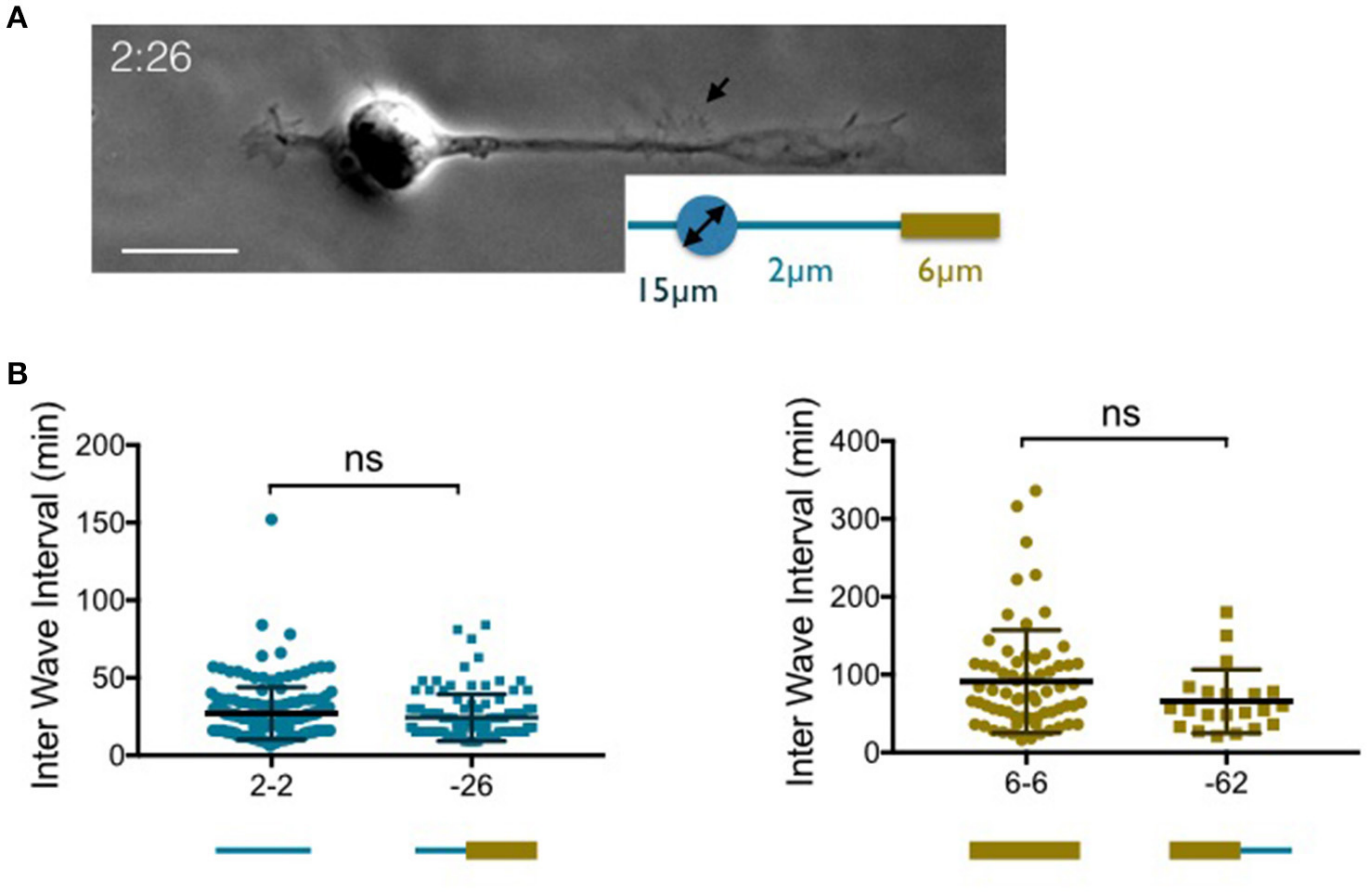
**Temporal patterns of waves according to the width of the neurite base. (A)** Images of a neuron grown on 2:26 adhesive pattern (see the inset) made of a 15 μm disk to anchor the soma and a 2 μm wide stripe that enlarges up to 6 μm on the right neurite 40 μm away from the edge of the soma. Scale bar: 15 μm. **(B)** Distribution of the time intervals between two successive actin waves along a given neurite (inter wave intervals: IWI) in different configurations where the width of the neurite base is either 2 μm (left) or 6 μm (right). Mean values and *SD*: 27.1 ± 16.8 min (2-2, *n* = 173, 4 cells) 24.3 ± 15.1min (−26, *n* = 105, 4 cells), 91.1 ± 65.9 min (6-6, *n* = 68, 6 cells) and 65.7 ± 40.8 min (−62, *n* = 21, 6 cells). ns: *p* = 0.21 (2-2 vs. −26) and *p* > 0.99 (6-6 vs. −62).

**Figure 3:**
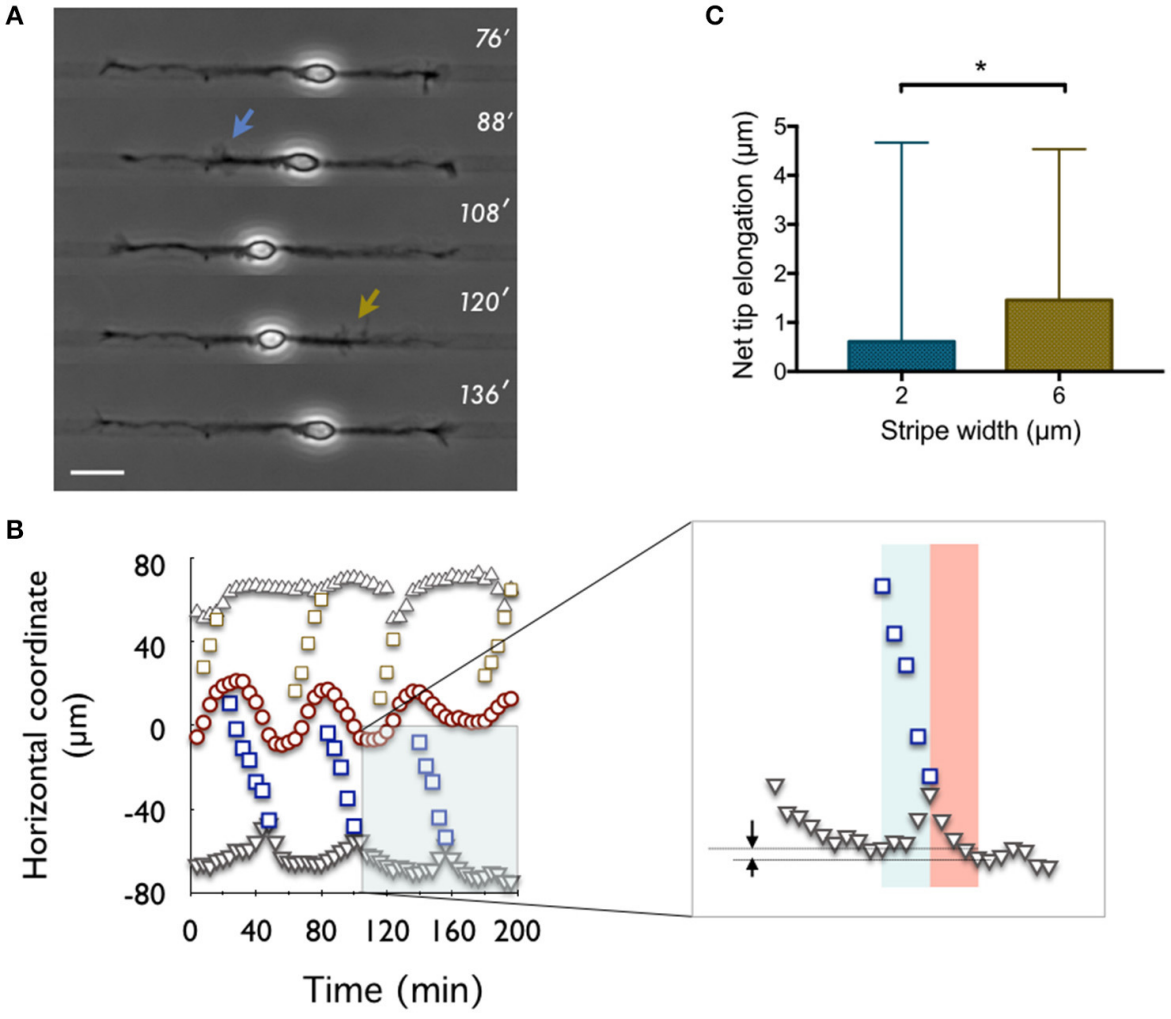
**Net tip elongation per actin wave. (A)** Time-lapse recording (indicated in minutes: 0′ is 24 h after plating) of a neuron developing on a 6 μm wide stripe (pattern 6-6). The adhesive pattern is revealed by phase contrast imaging as a dark stripe on a lighter gray non-adhesive background. Scale bar: 20 μm. **(B)** Coordinates of waves (open symbols), neurite tips (solid gray symbols), and soma center (red symbols) as a function of time for the neuron shown in **(A)**. The origin of the spatial coordinates is set to the initial soma position. Inset: zoomed region of the main graph. Arrows express the net tip growth that results from the sum of the initial tip retraction and the subsequent growth spurt computed within the blue and red windows, respectively. **(C)** Distribution of the net tip elongation per actin wave on 2 μm (blue) and 6 μm (gold) wide stripes, i.e., the 2-2 and 6-6 patterns, respectively. Mean values: 0.61 μm (2-2, *n* = 201, 5 cells) and 1.46 μm (6-6, *n* = 57, 5 cells). ^*^p = 0.0355.

**Figure 4:**
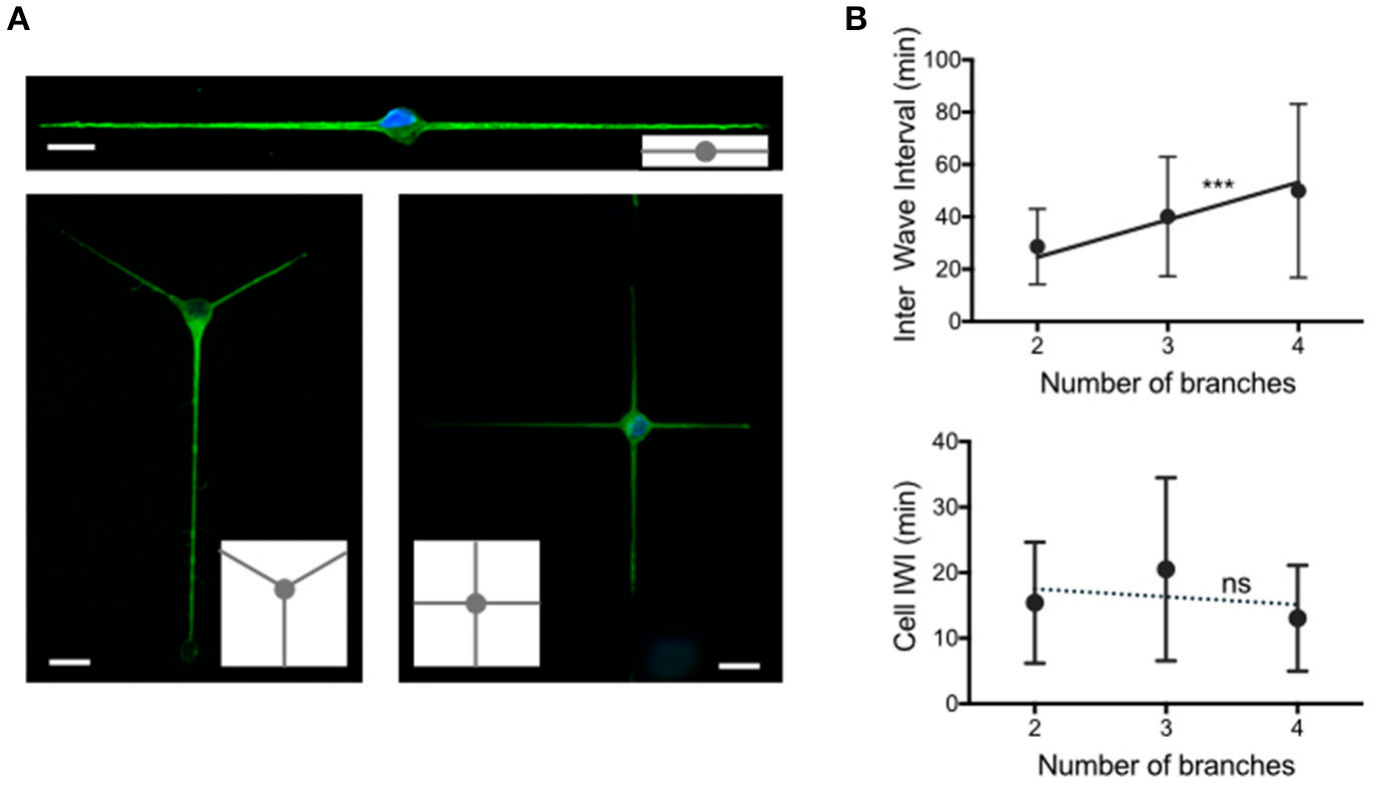
**Temporal patterns of waves according to the number of neurites. (A)** Images of 2 days *in vitro* neurons extending 2, 3, and 4 neurites grown on adhesive patterns made of 2 μm wide stripes and a central 15 μm diameter disk. Green: YL1/2, microtubules. Blue: Hoechst, nuclei. Adhesive patterns are represented in gray in the insets. Scale bars: 15 μm. **(B)** Distribution of the time intervals between two successive actin waves (Inter Wave Interval, IWI), computed at the neurite level (top) and at the cell (soma) level (bottom) for the three different configurations shown in **(A)** The solid line in the top graph of slope 14.3 min/branch constitutes the fit of our data following the hypothesis that actin waves are produced at a constant rate in the soma and distributed randomly along neurites (^***^p = 0.0005). Top graph mean values and *SD*: 28.6 ± 14.4 min (2 branches, *n* = 155, 5 cells), 40.1 ± 22.8 min (3 branches, *n* = 113, 2 cells) and 49.9 ± 33.2 (4 branches, *n* = 179, 3 cells). Bottom graph mean values and *SD*: 15.4 ± 9.2 min (2 branches, *n* = 166, 5 cells), 20.5 ± 14.0 min (3 branches, *n* = 115, 2 cells) and 13.0 ± 8.1 (4 branches, *n* = 188, 3 cells). The linear regression analysis of the data as a function of the stripe width is represented as a dashed line in the bottom graph (ns: *p* = 0.9).

Data were analyzed using Prism 7.0 (GraphPad Software, Inc.). Two-tailed unpaired non parametric Mann-Whitney U test were used for comparing IWI distributions (Figures 1, 2) and net tip elongation (Figure 3C). Chi-square tests were used for analyzing the slopes of the linear regression fits of the graphs of Figure 4. Distributions in Figures 1, 2, 4 are defined by their mean and standard deviation (*SD*) values.

### Patterning

Poly-L-lysine patterns were transferred on silanized substrates (18 × 18 mm^2^ coverslips from VWR, ref. 631–1331). After an oxygen plasma cleaning step (50W) of 2 min in duration, silanization was performed in a liquid phase using the 3Methacryloxypropyltrimethoxysilane (Bind-Silane, Sigma). Then, patterns were defined using UV classical photolithography steps, including Shipley S1805 photoresist spinning (4,000 rpm, 0.5 μm thickness, 115°C annealing step for 1 min), insulation through a mask, development (Microposit concentrate 1:1, Shipley), Poly-L-lysine deposition (1 mg/ml over night), and lift-off using an ultra-sound ethanol bath.

## Results

### Geometrical cues determine properties of actin waves

Diverse neuronal morphologies were produced from the design of various micropatterns of adhesion, in order to challenge the production of actin waves in situations involving e.g., different neurite widths, or different neurite number. More precisely, in a first set of experiments, we studied neurons with 2 neurites, on patterns in which each neurite was constrained to grow on either a 2 or 6 μm wide stripe, or on stripes with a varying width, from 2 to 6 μm and from 6 to 2 μm, respectively. In another set of experiments, we constrained neurons to grow on patterns inducing different numbers of neurites. Time-lapse experiments using phase contrast imaging were used to identify propagative actin waves (Fig. S1 and supplementary movie; See also the Materials and Methods Section).

#### The neurite width controls the production rate of actin waves

Following previous results showing that neurite elongation was slowed down when neurites were allowed to spread on 6 μm, as compared to 2 μm wide stripes (Tomba et al., 2014), we studied the temporal rate of actin wave production in similar geometries of adhesion. Linear adhesive patterns with widths of 2 or 6 μm, respectively, were produced, leading to 2-branched neurons displaying diametrically opposed neurites spreading on 2 or 6 μm wide stripes (these designs are referenced later on by the nomenclature 2-2 or 6-6, respectively). We selected young stage 2 neurons (Dotti et al., 1988), i.e., neurons of rather symmetric neurite length taken at 1–2 days *in vitro* (1-2 DIV) displaying a ratio between the longest and the shortest neurite lower than 1.5, except for transient and reversible neurite asymmetry due to soma displacement (see supplementary movie). Such an early stage of development is considered as the most relevant time window to assess the role of actin waves (Winans et al., 2016). We also designed a pattern combining diametrically opposed 2 and 6 μm wide stripes. A central disk of adhesion of 15 μm diameter was introduced in this asymmetric pattern to set the location of the soma and therefore the width of both neurites. In our nomenclature, this pattern is labeled “2:6,” with the mark “:” standing for the 15 μm disk. Figure 1A displays images of neurons grown on the three types of patterns, 2-2, 6-6, and 2:6, all exhibiting a single actin wave. It should be noted that, in the 2:6 patterns, we could not obtain well-formed 6 μm wide stumps before the 2 μm wide branch gets rather long, as illustrated by the cell shown in Figure 1A. This point is highlighted in Fig.S2A displaying the average neurite lengths for all conditions at 2 DIV and showing that 2:6 patterns inherently produce the most asymmetric neurons in length.

To evaluate the rate of actin wave initiation in these three geometrical configurations (i.e., 2-2, 6-6, and 2:6 patterns), we computed a quantitative characteristic, the Inter Wave Interval (IWI), corresponding to the time interval between the initiation of two consecutive actin waves within a given neurite. In the case of stripes with uniform width, data from both neurites of each of the analyzed cells were merged. For an overview of the way actin waves distribute temporally between the two branches, see Fig. S3 displaying the directional correlations between two subsequent actin waves.

The graph of Figure 1B summarizes our results. Strikingly, the mean interval between consecutive actin wave production events seems to scale with the pattern width. Actin waves are indeed about three times less frequent on wide neurites that elongate on 6 μm wide stripes, as compared to neurite growing on 2 μm wide stripes. This finding is further strengthened by the results obtained on the 2:6 patterns. We observed no significant differences between the IWI's distributions from the 2 μm wide branch of the 2:6 pattern and the distribution of IWIs obtained on 2 μm wide stripes (2-2 pattern). The same observation was made for the 6 μm wide branches. In other words, each branch of the 2:6 pattern behaved independently of the other: this suggests that actin waves are initiated according to a temporal pattern defined locally at the neurite level.

Actin waves travel distally along neurites but usually emerge near the soma. We therefore wondered if the width of the proximal segment alone was responsible for the observed distributions of IWIs. To answer this question, we designed variants of the previous patterns combining 2 and 6 μm wide stripes *along* a single branch (see Tomba et al., 2014). For instance, a 2 μm wide stump was inserted at the basis of a 6 μm wide stripe, yielding a geometry labeled as “−26” (see Figure 2A for an example of a neuron grown on a 2:26 pattern, according to the terminology defined above). The reverse situation, i.e., a 6 μm wide stump inserted at the basis of a 2 μm wide stripe was also used (and referenced as “−62”). Finally, to ensure that actin waves were initiated on stripes of uniform width near the soma, the lengths of the 2 or 6 μm wide proximal stumps were taken to be at least two times longer than the typical longitudinal actin wave dimension, i.e., about 10 μm (Figure 1A).

The graphs of Figure 2B show that there is no significant difference between the rate of actin wave production along neurites elongating on 2-2 and −26 stripes. The same observation was made between 6-6 and −62 geometries. This indicates that the geometrical control of the periodicity of actin waves is localized within the region of emergence of these structures near the soma.

#### The net growth triggered by the arrival of an actin wave at the neurite tip depends on the neurite width

It was reported in the seminal papers of Ruthel and Banker (Ruthel and Banker, 1999) that actin waves were triggering a retraction of the neurite tip while moving forward, then its extension. The combination of both phenomena resulted in a net forward growth of the tip. A similar observation was recently reported by Winans et al. (2016) who stated that, at 1 DIV, 90% of actin waves yield neurite outgrowth when reaching the tip, suggesting the central role of actin waves in the early stages of neuronal growth. Here, we computed the distributions of the net displacement of the neurite tip associated to actin waves produced on 2 and 6 μm wide stripes, respectively (only actin waves that could be followed up to the tip where taken into account). In all the experiments selected, the onset of actin waves and their position during their propagation were measured (Figures 3A,B). The progressive retrograde motion of the tip seemed well correlated with the time span of these propagative structures. Defining the duration of the extension phase was less straightforward. We noted however that similar time constants seemed to be involved in the retraction and extension processes, as illustrated by the rather symmetric shapes of the corresponding peaks in the curves representing the tip position versus time (Figure 3B). We thus decided to compute the tip extension during a time span equal to the duration of its retraction, as expressed by the identical widths of the red and blue rectangles in the inset of Figure 3B. Other criteria could have been chosen, e.g., to wait for a stabilization of the position of the tip consecutive to the burst of growth triggered by the actin wave, but then our observation would have depended strongly on the time elapsed between two successive actin waves on the same neurite. The relatively short duration chosen to compute the neurite extension phase might consistently lead to an underestimation of the total neurite elongation. However, our procedure should provide a precise proxy for neurite elongation that allows a comparison between different adhesive patterns.

Using the above procedure, we found that the increment of growth per actin wave λ_*w*_ was larger for 6 μm wide than for 2 μm wide stripes, i.e., λ_6_ = 1.46 μm (*n* = 57, 5 cells) and λ_2_ = 0.61 μm (*n* = 201, 5 cells), respectively, as illustrated in Figure 3C. Note that previous work reported larger values of net growth in the range of 2–4.5 μm per wave, using a longer time window at the expense of a less clearly defined criterion (Ruthel and Banker, 1999). But even in the presence of a proportional measurement bias, as described just above, the ratio between these numbers should correctly quantify the ratio of growth induced by the waves on each of these stripes.

#### The production rate of actin waves by the cell is independent of the number of neurites

The above experiments suggest a local control of actin wave production, at the neurite basis. In order to test this observation in a different context, we explored a configuration in which the neurite width is set at a fixed value (2 μm), and the number of neurites varies. We therefore designed adhesive geometries with a central disk (diameter 15 μm) and 2 μm thin lines symmetrically distributed around this disk in order to produce neurons with 2, 3, or 4 branches (see Figure 4A for the sketches of the adhesive patterns and images of typical cells).

The distribution of IWI computed in two different ways, i.e., at the neurite and cell levels, similarly to Figure 1, 2, respectively), is reported in Figure 4B for each geometry. Interestingly, the mean values of IWI at the neurite level increases nearly proportionally with the number of branches, whereas the global rate of production of actin waves computed at the cell level does not vary significantly with the number of branches. Note that this invariance of the mean IWI computed at the scale of the entire neuron versus the number of branches is associated with a conservation of the total neurite length at 2 DIV (Fig. S2B).

### Modeling neuron growth and axonal polarization using the geometrical determinants of actin waves

In a previous work, we had studied the influence of patterns geometry on neurite length at 3DIV, and on the localization of axonal specification [see Tomba et al. (2014), and the supplementary material of this work]. Basically, we used in (Tomba et al., 2014) 4 different types of 2 branch patterns built from 2 and 6 μm wide adhesive stripes. As an example, a pattern defined by a 6 μm wide stripe on one side of a 15 μm-diameter disk, and a diametrically opposed 2 μm wide stump that will further be allowed to enlarge to up to 6 μm after a length l will be defined as a 6:26 pattern. Similarly, we produced 6:62, 2:26, and 2:62 patterned neurons with l = 20, 40, 60, and 100 μm except for the 2:62 pattern (where l = 10 and 30 μm). The results could be interpreted by a phenomenological model, in which the tip was assumed to grow at a speed defined by the local stripe width at the tip's location, and by the polarization state of the neurite (dendrite or axon). All the experiments were accounted for by a single set of parameters of the model—i.e., the critical length that sets the axonal fate, the width of the critical length distribution and the width and polarization dependent growth rates. This model, however, did not provide insight in the mechanisms responsible for these behaviors.

#### Actin-wave based model of neurite growth and polarization

In the present work, we used our findings about the geometrical determinants of actin waves obtained from simple patterns to build a new mechanistic model of neuronal growth and polarization, assuming that these growth and polarization and mainly driven by actin waves. This model was then compared with the experimental data previously obtained at 3 DIV on the more complex sets of patterns described above.

More specifically, we modeled the elongation of the neurites along the adhesive stripes from the following assertions, motivated by the accumulated experimental observations:

- Growth velocity is affected by the rate of actin-wave initiation, which in turn is determined by the proximal width (close to the soma) of the adhesive stripe on which the undifferentiated neurite is growing. This velocity is obtained from the product of the width-dependent actin wave mean initiation rate (Figure 1) by the length increment of the tip induced per actin wave (Figure 3D).
- Neurites have a certain probability to turn into axons, which signals for the whole cell to polarize. In the model, this polarization event will occur when the neurite length exceeds a critical length, the distribution of which is assumed as Gaussian. This neurite will then differentiate into an axon, while all other neurites will then become dendrites. This part of the model, motivated by previous studies in this field (Seetapun and Odde, 2010; Yamamoto et al., 2012), is unchanged from (Tomba et al., 2014).
- The growth velocity of the differentiated neurites will follow the change in the mean rate of actin wave initiation induced by the event of axonal determination, i.e., increase in the axon and decrease in the dendrites.

These assertions are mathematically expressed as described below.

The average growth velocity *v* of the neurite tip in condition of controlled neuronal geometries is ruled by the following set of equations:

(1) $$v_{w}={\alpha \lambda }_{w}\omega_{w}$$

where ω_*w*_ is the production rate of actin waves on an initial stripe segment of width *w*, λ_*w*_ the growth increment per actin wave and α a free parameter expressing the proportional measurement bias discussed in section 2.2.

In addition, this velocity *v*_*w*_ will change by a concentration/dilution factor θ, if the growing tip has moved past a stripe width change, to account for the conservation of the material conveyed by actin waves, i.e.,

(2) $$v_{w}={\alpha \lambda }_{w}\omega_{w}\theta$$

θ>1 (resp. θ < 1) when the actin wave passes from a wide (resp. thin) region to a thinner (resp. wider) region, so that the material delivered to the leading edge at the tip is concentrated (resp. diluted) and therefore gives a larger (resp. smaller) net growth.

Following polarization, the equations displayed below will rule the growth of axons and of future dendrites:

(3) $$\begin{array}{l} v_{w\;(axon)}=\beta v_{w\;(neurite)},\\ v_{w\;(dend.)\;}=\gamma v_{w\;(neurite)} \end{array}$$

Experimentally, β and γ express the change in actin wave initiation mean rate following the event of axonal determination in our present model, as addressed in the following section.

#### Experiments specifying the parameters of the model

First, we model *L*_*pol*_ by a Gaussian distribution with mean *L*_*pol*_ = 50 μm and standard deviation σ_*pol*_ = 20 μm, as already considered in the work of Tomba et al. (2014). This choice matches with the observed length of the initial axonal segment, a structure implied in action potential generation.

The set of parameters associated with actin waves are the following: α, ω_*w*_, λ_*w*_, θ, β, γ with *w* = 2 or 6 μm. We have already experimentally determined ω_*w*_ and λ_*w*_ for both values of *w* (see Figures 1, 3, respectively).

To determine β and γ, we seek the rates of production of actin waves in the nascent axon and in the future dendrite, as compared to the rate obtained in undifferentiated, symmetric neurons (cf. the data shown in Figure 1). We selected 1 DIV neurons with already well-developed symmetric neurites and followed them up to 2 DIV (see Fig. S4A). Interestingly, we observed no obvious change in the periodicity of actin waves at the soma level when neurons evolve from a symmetric to an asymmetric morphology evoking a transition to a polarized state (see Fig. S4B). This observation suggests a constraint on β and γ, such that $\frac{\gamma +\beta }{2}=1$.

To go further, we then considered another set of patterned neurons on stripes characterized by asymmetric lengths (with one neurite at least 50% longer that the other) and already long neurites (> 50 μm), so that one neurite should have differentiated into an axon. We measured the IWI of each branch and found a mean value of $\frac{\beta }{\gamma }=r\approx 2.2\;\pm \;0.57$, meaning that axons convey roughly twice as many actin waves as dendrites (Fig. S5).

Combined with the relation γ+β = 2, this yields: $\gamma =\frac{2}{1+r}\approx 0.6$, β = *rγ* ≈ 1.4.

Finally, we expect the dilution factor θ to be at most equal to the ratio $\frac{w_{i}}{w_{f}}$ of the widths before/after the transition region, as the material is delivered and spread over the leading edge that spans the stripe width. In our model, θ will remain a free fitted parameter. Similarly, the value of the parameter α that tunes the absolute value of neurite growth is also an adjustable parameter of the model.

#### Results of the model

**Figure 5:**
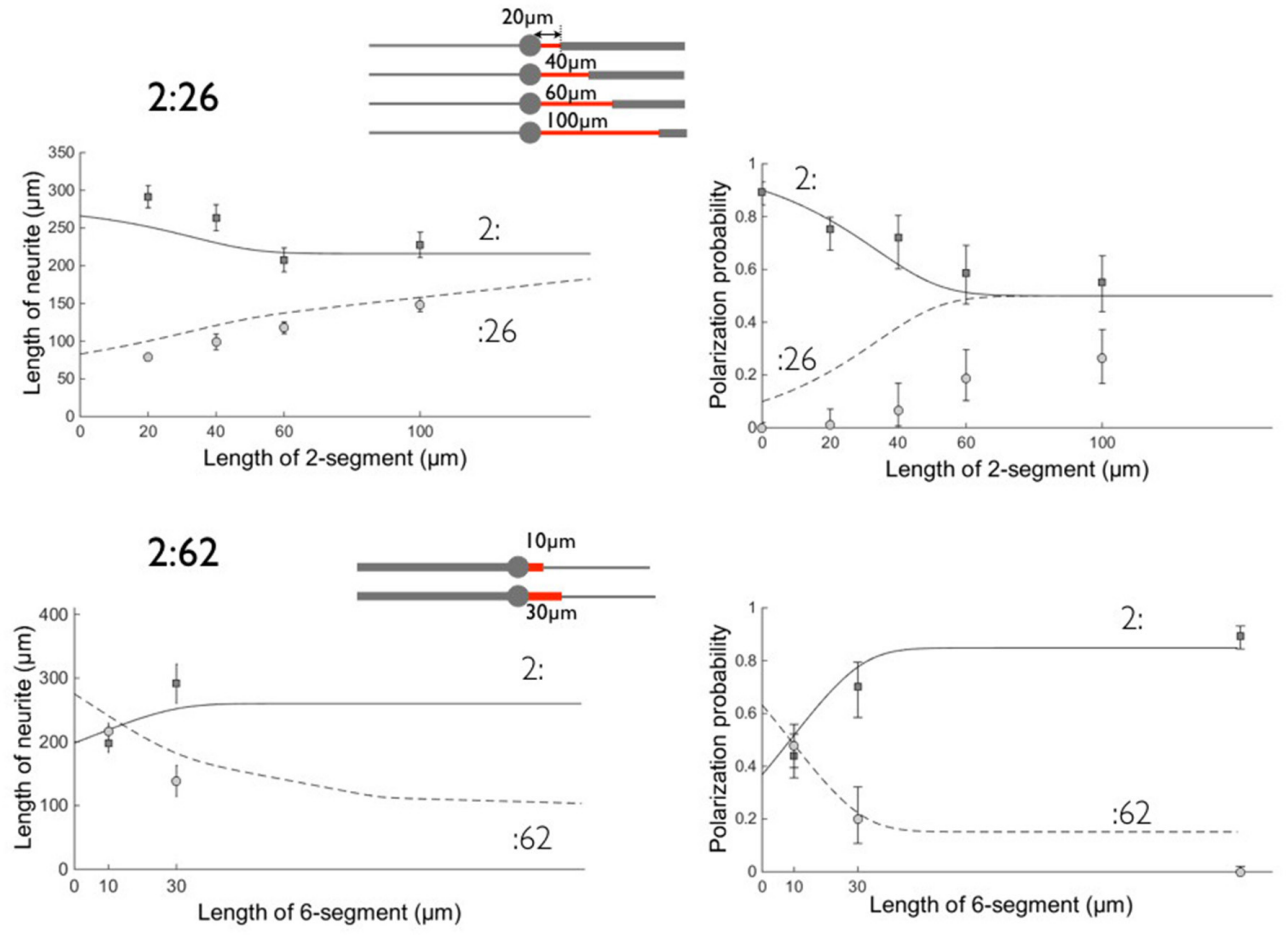
**Comparison between experimental data and the model on patterns 2:26 and 2:62. Left**: neurite lengths, **right:** polarization probabilities. All results were obtained at 3 days *in vitro*. Symbols denote the experimental data and lines the result of the model calculation: Circles (dashed line) correspond to the :26 and :62 branches and squares (solid line) to the :2 opposite branch. Errors bars represent the standard error of the mean (length data) or 95% confidence interval (polarization data). The parameters used in this calculation are: *T* = 72 h, *v*_2_ = α λ_2_ω_2_ ≈ 3μm/hr, *v*_6_ = α λ_6_ω_6_ ≈ 1.8μm/h (α = 2.2), β = 1.4, γ = 0.6, θ = 2. As previously (see Tomba et al., 2014), we took *L*_*pol*_ = 50 μm, σ_*pol*_ = 20 μm, and we inserted polarization data obtained on 2:6 patterns (we located them arbitrarily at a coordinate l = 150 μm for 2:62 data). The schemes of the patterns are shown for each set of geometries with the proximal stump of variable length in red.

**Figure 6:**
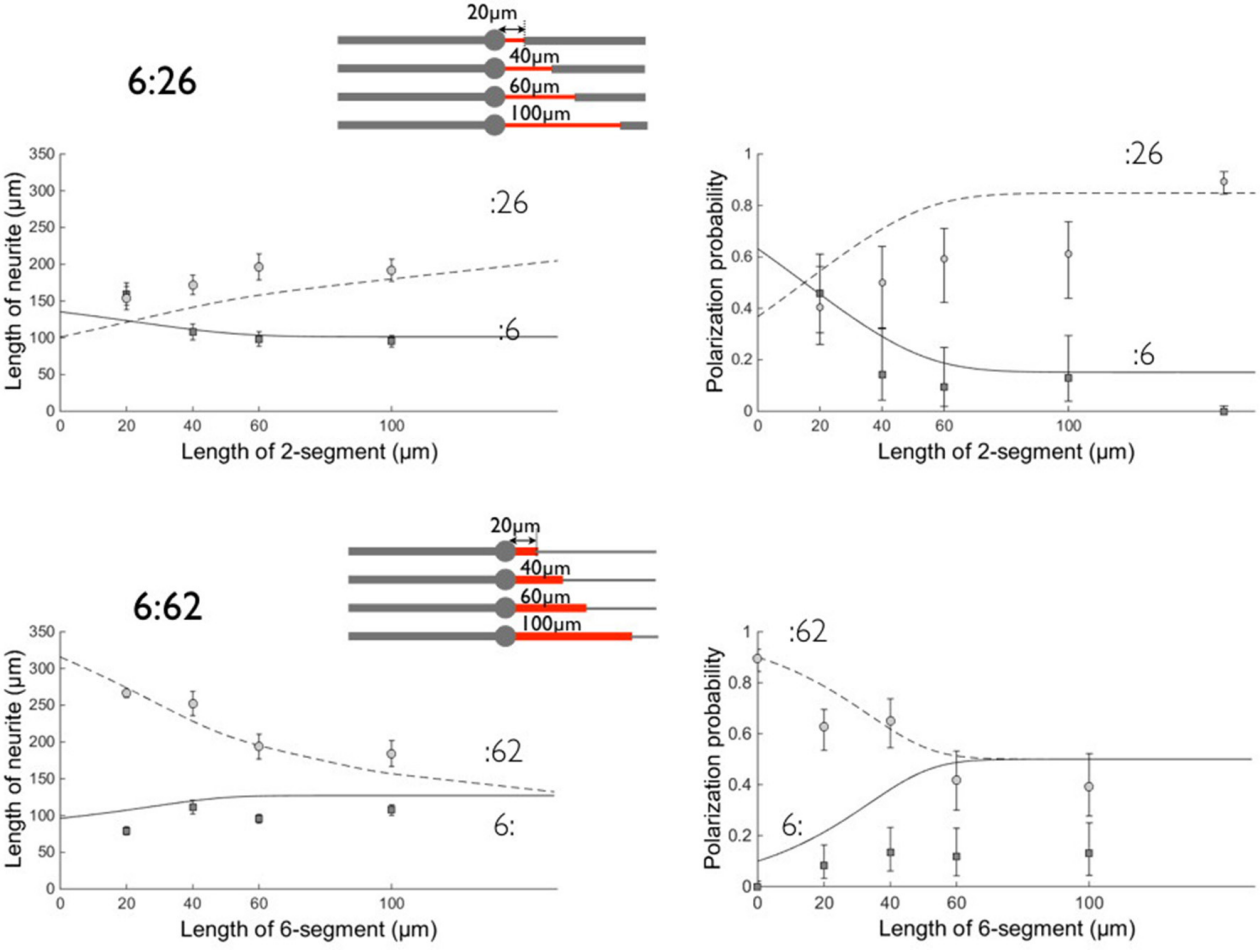
**Comparison between experimental data and the model on patterns 6:26 and 6:62**. Left: neurite lengths, right: polarization probabilities. All results were obtained after 3 days of growth. Symbols denote the experimental data and lines the result of the model calculation (see Figure 5). Circles (dashed line) correspond to the :26 and :62 branches and squares (solid line) to the :6 opposite branch. Errors bars represent the standard error of the mean (length data) or 95% confidence interval (polarization data). Same values of fit parameters as in Figure 5. Polarization data obtained on 2:6 patterns are, as in Figure 5, located arbitrarily at a coordinate l = 150 μm in the 6:26 graph. The schemes of the patterns are shown for each set of geometries with the proximal stump of variable length in red.

The data of Tomba et al. (2014) were used to challenge our new model. We used the above numbers deduced from the experiments of the present work. In addition, we let θ (i.e., the dilution/concentration factor of the wave material when the neurite width changes) and α (i.e., the width-independent coefficient regarding the amplitude of the tip net growth induced by an actin wave) as the only two free parameters. The comparison between the model and the experimental data is shown in Figures 5, 6. Note that axons were identified through Tau-1 immunolabelling (see Fig. S6 for a few examples of stained neurons).

## Discussion

As previously, the model gives higher polarization probabilities than experiments, in particular when the Tau-1 labeling should be observed on 6 μm wide stripes. Following (Tomba et al., 2014), we associated this discrepancy with a possible impaired establishment of the Tau-1 gradient under this condition of unusual neurite spreading.

We found reasonable fits using θ = 2 for the concentration factor when the waves move from 6 to 2 μm wide stripes (and 1/ θ for the dilution factor in the symmetric case). Although lower than expected from the ratio $\;\frac{w_{i}}{w_{f}}=3$, this value lies in the range 1 < θ < 3, expressing the existence of an effective concentration/dilution effect according to our model. We also found α ~ 2.2, i.e., a bare growth velocity along the 2 μm stripe of 3 μm/h. This value α >1 supports the view that we probably have underestimated the values of λ_*w*_ by choosing an arbitrary time window over which to compare the net forward growth per waves on 2 and 6 μm wide stripes. This value of α might also reflect the fact that lengths were computed at 3 DIV in order to retrieve polarization data, i.e., at a stage in which actin waves might have a lower contribution to neuronal growth (Winans et al., 2016).

Notwithstanding the above question, however, we emphasize that with only two free parameters, our model consistently accounts for different sets of experiments using a variety of patterns. In addition, it successfully captures a qualitative feature of the experiments that was not covered by the previous model of Tomba et al. (2014), i.e., the crossing of the curves associated with the length and polarization probabilities of the two branches of the 2:62 and 6:26 patterns versus the length *l* of the proximal stump. For example we found that, for a 20 μm long 6 μm wide stump of the 2:62 patterns, the :62 branch was experimentally the longest one (i.e., longer than the :2 branch). From our present model, this situation arises from the association of three phenomena that boost the growth velocity of the −62 branch from the very beginning: (i) the 6 μm wide short segment delivers an actin wave with a high quantity of material, i.e. 2.4 times higher than delivered on the 2 μm wide branch; (ii) this material is further concentrated into the remaining 2 μm wide portion of the neurite, boosting its growth by a factor θ = 2 and making it win the competition for polarization by reaching the critical length before the other (2 μm) branch; (iii) following its differentiation into an axon, the :62 branch undergoes an even faster growth. This advantage in favor of the :62 branch is lost for longer 6 μm wide portions, since the growth velocity is then dominated by the λ_2_ω_2_ term during a time window long enough to allow the 2 μm wide branch to undergo axonal polarization. For the same reasons, the competition between the 6: and the :26 branches is biased in favor of the 6: branch, when the 2 μm wide stump is short: this is a consequence of the dominant role of the dilution effect at the :26 transition, combined with the lower amount of materials transported by actin waves in this configuration. Note that our model takes into account the effect of the proximal segment even if its length *l* goes to zero. The longitudinal extension of actin waves should impose a cut-off of the order of 10 μm (i.e., the longitudinal extension of an actin wave) below which our assumptions and model are no longer valid.

In addition, the same values of the modeling parameters (except for θ which was not needed anymore) were used to model the results obtained at 2 DIV on the 2-3-4 patterns built from 2 μm wide stripes (see also Fig. S2A for results regarding neurite length associated to 2-3-4 branches patterns). In these calculations, we assumed that the initiation rate per neurite is diminished by the factor 1/(number of branches), to account for the conservation of actin-wave initiating material at the soma. Fig. S7 shows a very reasonable fit of our experimental data, which constitutes an additional support for our description of the role of actin waves in neuronal growth.

Lastly, although the model presented in this work is based on the consequences of the initiation and propagation of actin waves and does not deal with the microscopic mechanisms behind these phenomena, we made a few observations that may feed a future mechanistic model of actin waves. In Figures 3A,B we observed, as did previous authors (Ruthel and Banker, 1999; Winans et al., 2016), that actin waves were pulling on the neurite tip. Due to the limited surface of adhesion provided to the soma by adhesive stripes, we also observed a nucleokinesis phenomenon associated with the propagation of actin waves. Actin waves seem therefore to behave like propagative contractile nodes exerting tension between the soma and the tip. This observation, and the contribution of actin waves in neurite elongation emphasized in the present work, echoes existing literature about the role of mechanical forces and neurite tension in neuronal growth (Zheng et al., 1991; Chada et al., 1997; Lamoureux et al., 2002). This suggests that, besides the supply of membrane and other materials provided by actin waves (Ruthel and Banker, 1999; Flynn et al., 2009; Toriyama et al., 2006), these structures also contribute to the generation of mechanical forces inside neurites. Indeed, we observed that poly-lysine functionalized polystyrene beads attached to the neurite membrane were systematically transported in retrograde fashion to the soma (data not shown). Such an observation was already reported in DRG neurons, and attributed to plasma membrane flow due to differential membrane tension between the growth cone and the soma (Dai and Sheetz, 1995). How this retrograde flow would be connected to the anterograde transport provided by actin waves and would contribute to build membrane tension is an exciting question that we hope will motivate further studies.

## Conclusion

In this work, we provide a quantitative characterization of the generation of actin waves in different neuronal morphologies, efficiently modulated by the use of micropatterns of adhesion. We found that the rate of production of actin waves was independent on the number of neurites, suggesting that the material conveyed by actin waves is produced at the soma level. In addition, our finding that the rate of production of actin waves scales with the proximal neurite width might suggest that this material is stochastically distributed at the neurite base until it reaches the threshold required to build a propagative actin wave. Therefore, micropatterns of adhesion proved to be very fruitful to dissect and reveal the properties of actin waves in controlled conditions. In addition, they provided a valuable tool to test, on a large set of various neuronal morphologies, a model built using simple assumptions about the geometrical determinants of actin waves.

We hope that these novel results regarding actin waves will contribute to their understanding and orient future molecular and biomechanical studies of these propagative structures.

## Author contributions

CT and CV designed the experiments and analyzed the data. CT, CB, and FC did neuron cultures and time-lapse experiments. GB contributed to the exploratory phase of experiments leading to this work. NG did the modeling and BF contributed to it. BF, NG, CT, and CV wrote the paper.

## Funding

This work was supported in part by the European Research Council Advanced Grant No. 321107 “CellO,” ANR Investissement d'Avenir, and the IPGG Labex and Equipex. N.S.G. is the incumbent of the Lee and William Abramowitz Professorial Chair of Biophysics.

### Conflict of interest statement

The authors declare that the research was conducted in the absence of any commercial or financial relationships that could be construed as a potential conflict of interest.
