# Supplementary material for: Geometrical Determinants of Neuronal Actin Waves
Source: Front Cell Neurosci. 2017 Mar 29;11:86. doi: 10.3389/fncel.2017.00086 (PMC5372798; doi:10.3389/fncel.2017.00086)
Supplement: Supplementary file 2 [file Presentation1.pdf]

Supplementary material : explanation of the model (from [Tomba2014])

We model the elongation of the neurites along the adhesive strips, by considering the following observations :

1. The growth velocity is affected by the width of the adhesive stripe on which the neurite is growing. We will consider the simplest scenario where the growth material synthetized at the soma level has to be distributed along the available surface at the neurite growth-cone, leading to a larger (smaller) velocity v_{tip} when the width is smaller (wider).
2. The neurites have a certain probability to turn into axons, which signals for the whole cell to polarize. This polarization event seems to depend on the absolute length of the neurite.
3. Following the polarization event the neurite that was "chosen" to become the axon may continue to grow at a different growth velocity βv_{tip} (with $\beta \geq 1$). The other neurite(s) continues to grow at a reduced growth velocity γv_{tip} ($0 \leq \gamma \leq 1$), following the polarization event.

We now turn these assumptions into a calculation of the mean neurite length on the patterns (denoted x :xy and x :yx, i.e. 2 :26, 6 :62, 2 :62 and 6 :26 as described in the main text) For this we need to assume some probability density function that describes the probability for the neurite to undergo polarization, as a function of its length. Based on the notion of a critical length for this transition, we consider the following simple step-like cumulative probability to polarize

$$P_{pol}(x) = \frac{1}{2}\left(1 + \tanh\left[\frac{x - L_{pol}}{\sigma_{pol}}\right]\right) \tag{1}$$

$$\Rightarrow p_{pol}(x) = \frac{dP_{pol}(x)}{dx} = \frac{1}{2\sigma_{pol}}\text{sech}\left[\frac{x - L_{pol}}{\sigma_{pol}}\right]^2 \tag{2}$$

where L_{pol} is the critical polarization length and σ_{pol} gives the variance of the probability distribution function around this critical length.

We then divide the calculation into two cases, for the case of the right neurite polarizing, and for the left. We now demonstrate the calculations that result from our model for the x :xy patterns. A similar calculation follows along the same lines for the x :yx patterns. For polarization along the right neurite, the length of the right neurite is given by the following

$$L_{R,R} = \begin{cases} x_{pol} + (T - t_{pol,1})\beta v_y, & [x_{pol} > l] \\ l + (T - t_l)\beta v_y, & [x_{pol} < l, T > t_l] \\ l + (T - t_{pol,2})\beta v_x, & [x_{pol} < l, T < t_l] \end{cases} \tag{3}$$

where l is the length of the x-segment on the right side, x_{pol} is the location of the polarization event on the right neurite, T is the time at which the observation is performed and $v_{x,y}$ are the tip velocities on the respective width. The different times are defined as

$$t_{pol,1} = \frac{l}{v_x} + \frac{x_{pol} - l}{v_y} \tag{4}$$

$$t_{pol,2} = \frac{x_{pol}}{v_x} \tag{5}$$

$$t_l = t_{pol,2} + \frac{l - x_{pol}}{\beta v_x} \tag{6}$$

$$\tag{7}$$

The maximal value of x_{pol} for the right neurite is

$$x_{pol,max,R} = \begin{cases} l + (T - (l/v_x))v_y, & [T > (l/v_x)] \\ Tv_x, & [T < (l/v_x)] \end{cases} \tag{8}$$

Over the possible range of x_{pol} we need to integrate the lengths in Eq.3 multiplied by the probability that the right neurite polarizes at position x_{pol}, which is given by $p_{pol}(x)$ (Eq.2). In addition we need to multiply by the probability that the competing neurite has not already polarized itself by this time. This probability is given by the cumulative probability up to the position of the tip on the left neurite $x_{pol,L}$ corresponding

to x_{pol} on the right neurite

$$\begin{aligned} p_{nopol,L}(x_{pol}) &= 1 - \int_0^{x_{pol,L}} p_{pol}(x)dx \\ &= 1 - \frac{1}{2}\left(\tanh\left[\frac{L_{pol}}{\sigma_{pol}}\right] - \tanh\left[\frac{L_{pol} - t_{pol}v_x}{\sigma_{pol}}\right]\right) \end{aligned} \tag{9}$$

where t_{pol} stands for $t_{pol,1}, t_{pol,2}$ given in Eqs.4,5 respectively, and $x_{pol,L} = t_{pol}v_x$.

The final mean length that we get is

$$\langle L_{R,R}\rangle = \int_0^{x_{pol,max,R}} L_{R,R}p_{pol}(x_{pol})p_{nopol,L}(x_{pol})dx_{pol} \tag{10}$$

where the overall probability for polarization of the right neurite is given by

$$P_R = \int_0^{x_{pol,max,R}} p_{pol}(x_{pol})p_{nopol,L}(x_{pol})dx_{pol} \tag{11}$$

For completeness, the overall probability for polarization of the left neurite is given by

$$P_L = \int_0^{x_{pol,max,L}} p_{pol}(x_{pol})p_{nopol,R}(x_{pol})dx_{pol} \tag{12}$$

where : $x_{pol,max,L} = Tv_x$, and

$$p_{nopol,R}(x_{pol}) = 1 - \int_0^{x_{pol,R}} p_{pol}(x)dx$$

$=$

$$\begin{cases} 1 - \frac{1}{2}\left(\tanh\left[\frac{L_{pol}}{\sigma_{pol}}\right] - \tanh\left[\frac{L_{pol}-x_{pol}}{\sigma_{pol}}\right]\right), & [l > x_{pol}] \\ 1 - \frac{1}{2}\left(\tanh\left[\frac{L_{pol}}{\sigma_{pol}}\right] - \tanh\left[\frac{L_{pol}-(l+(x_{pol}-l)*v_y/v_x)}{\sigma_{pol}}\right]\right), & [l < x_{pol}] \end{cases}$$

In a similar manner we calculate the length of the left neurite when the right one is polarized ($\langle L_{L,R}\rangle$) and the lengths for the case of polarization on the left neurite (i.e. $\langle L_{L,L}\rangle, \langle L_{R,L}\rangle$). In order to get the total mean length along each side, we also need to consider the lengths of the neurites when no side has polarized. For the $x : xy$ these lengths are given by

$$L_{L,0} = Tv_xP_{nopol} \tag{13}$$

and

$$L_{R,0} = \begin{cases} (l + (T - (l/v_x))v_y)P_{nopol}, & [T > (l/v_x)] \\ Tv_xP_{nopol}, & [T < (l/v_x)] \end{cases}$$

where the probability for no polarization is given by : $P_{nopol} = 1 - P_R - P_L$, where P_R is given in Eq.11.

Summing the lengths on each neurite from all the possible polarization outcomes gives the final mean lengths : $L_L = L_{L,0} + \langle L_{L,R}\rangle + \langle L_{L,L}\rangle$ and $L_R = L_{R,0} + \langle L_{R,R}\rangle + \langle L_{R,L}\rangle$.

Fig. S1

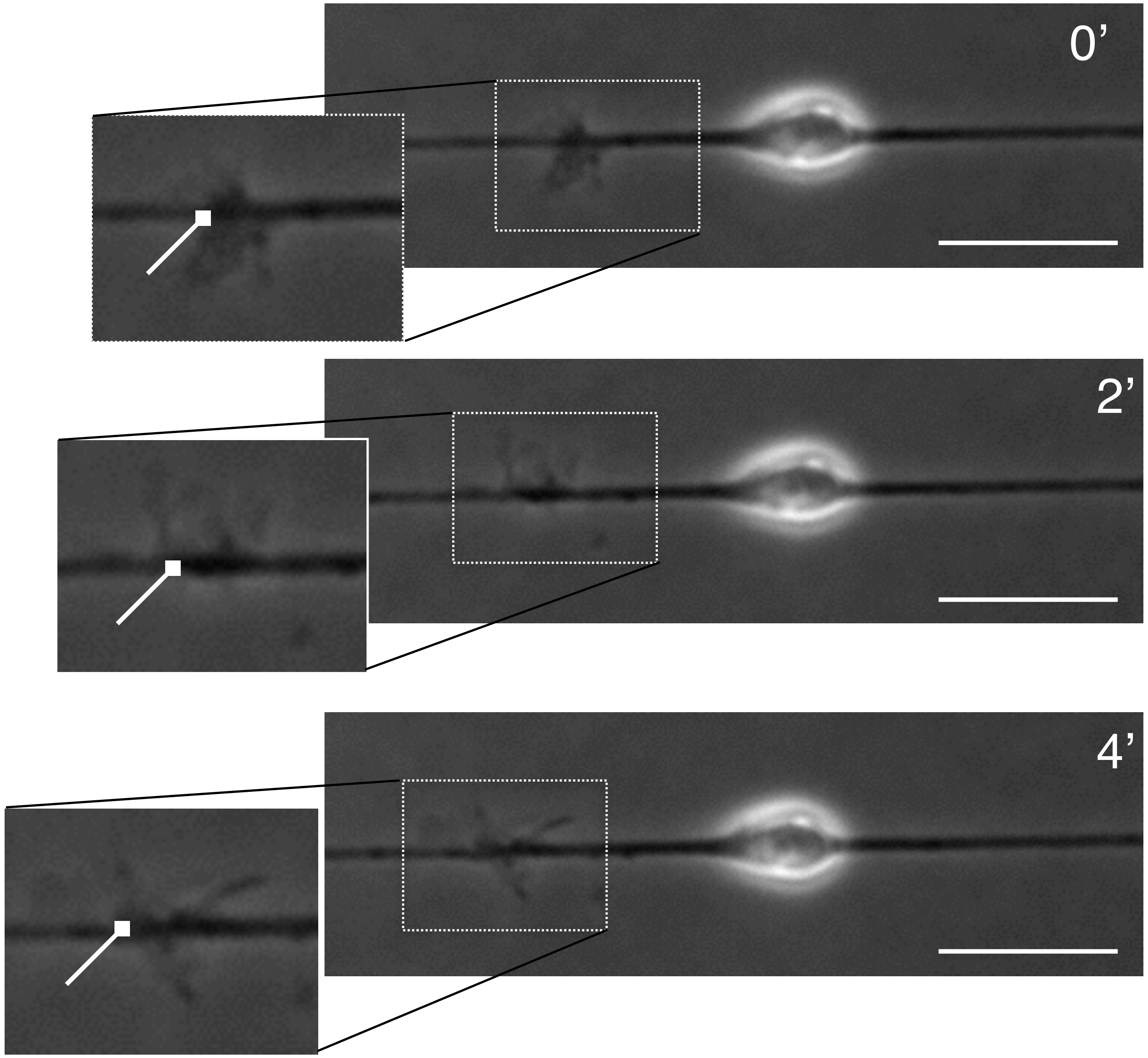

Fig. S1 - Criteria for the identification and analysis of actin waves. Consecutive frames of a neuron grown on a 2µm wide stripe. Zoomed images give a focus on an actin wave propagating on the leftward neurite. The caps of the white lines point the successive horizontal coordinates of actin waves, determined in our work from the boundary between a relatively dense and central area and the lamellipodium-like structure, more transparent, at the front of the structure. Time in minutes. Scale bar: 20µm.

Fig. S2

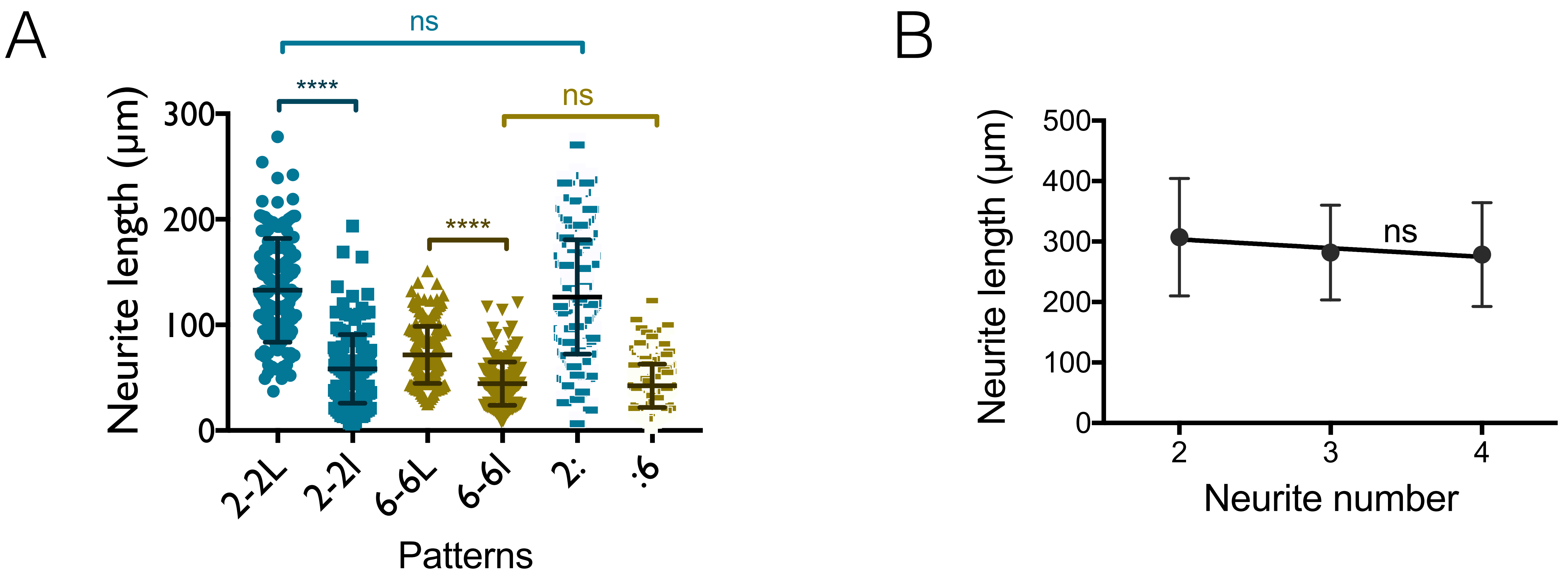

Fig. S2 - Distributions of neurite lengths. A - Distribution of neurite lengths on 2 and 6µm wide stripes at 2DIV. x-xL and x-xl indicate the length of the longest and shortest neurite, with x = 2 or 6µm. The 2: and 6: legends refer to the 2µm and 6µm wide stripes of the asymmetric 2:6 pattern. ****: p<0.0001; ns: p>0.9999. Dunn's multiple comparison tests. **B -** Distribution of total neurite length for neurons grown on patterns exhibiting two, three and four 2µm wide stripes radiating from a central 15µm disk dedicated to soma adhesion (see Fig. 3). The solid line represents the linear fit of the data. A chi-square tests was used for analysing the slopes of the linear regression fit . ns:p=0.27.

Fig. S3

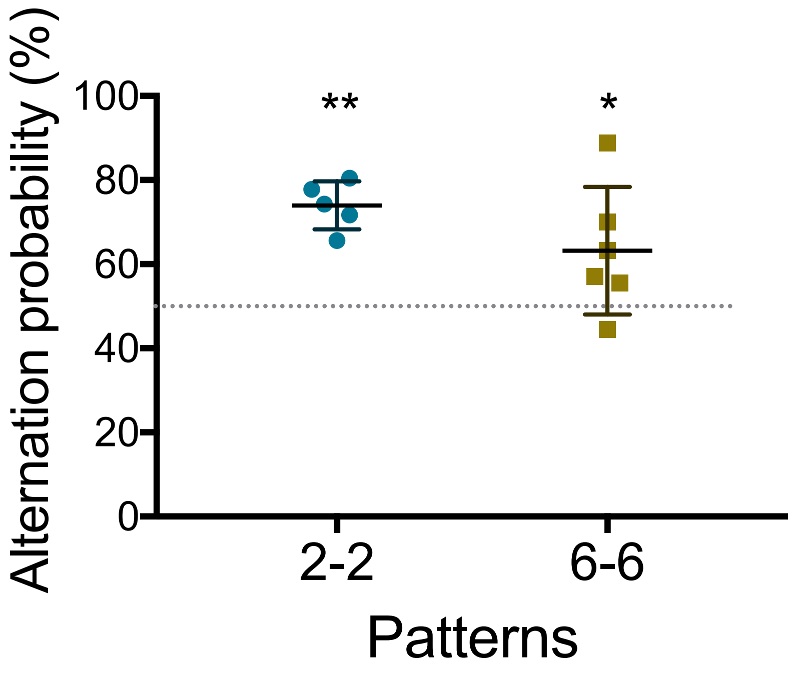

Fig. S3 - Repetition probability. A - After identification of individual neurites as left and right, we note N(Ss) the numbers of successive pairs of actin waves on the same neurite, *i.e.* “left following left” and “right following right” events. We similarly defined the number of alternative actin waves N(Al) as “left following right” and “right following left” events.
We then defined the “alternate repetition probability”as $P = \frac{N_{Al}}{N_{Al} + N_{Ss}}$

The repetition probability would be 50% in case of random shedding, and 100% in case of perfect alternative shedding. In bipolar neurons developing on 2µm (blue symbols) and 6µm (gold symbols) wide stripes, the experimental values of *P* are 74.0±5.7% and 63.2±15.2%, respectively, both significantly different from random. These values confirm a preferential alternative actin waves distribution, although this phenomenon is much accentuated for the thinner stripes.
**, p=0.008, n=296, 5 cells and *, p=0.047, n=90, 6 cells. Two-tailed unpaired non parametric Mann-Whitney U test were used.

Fig. S4

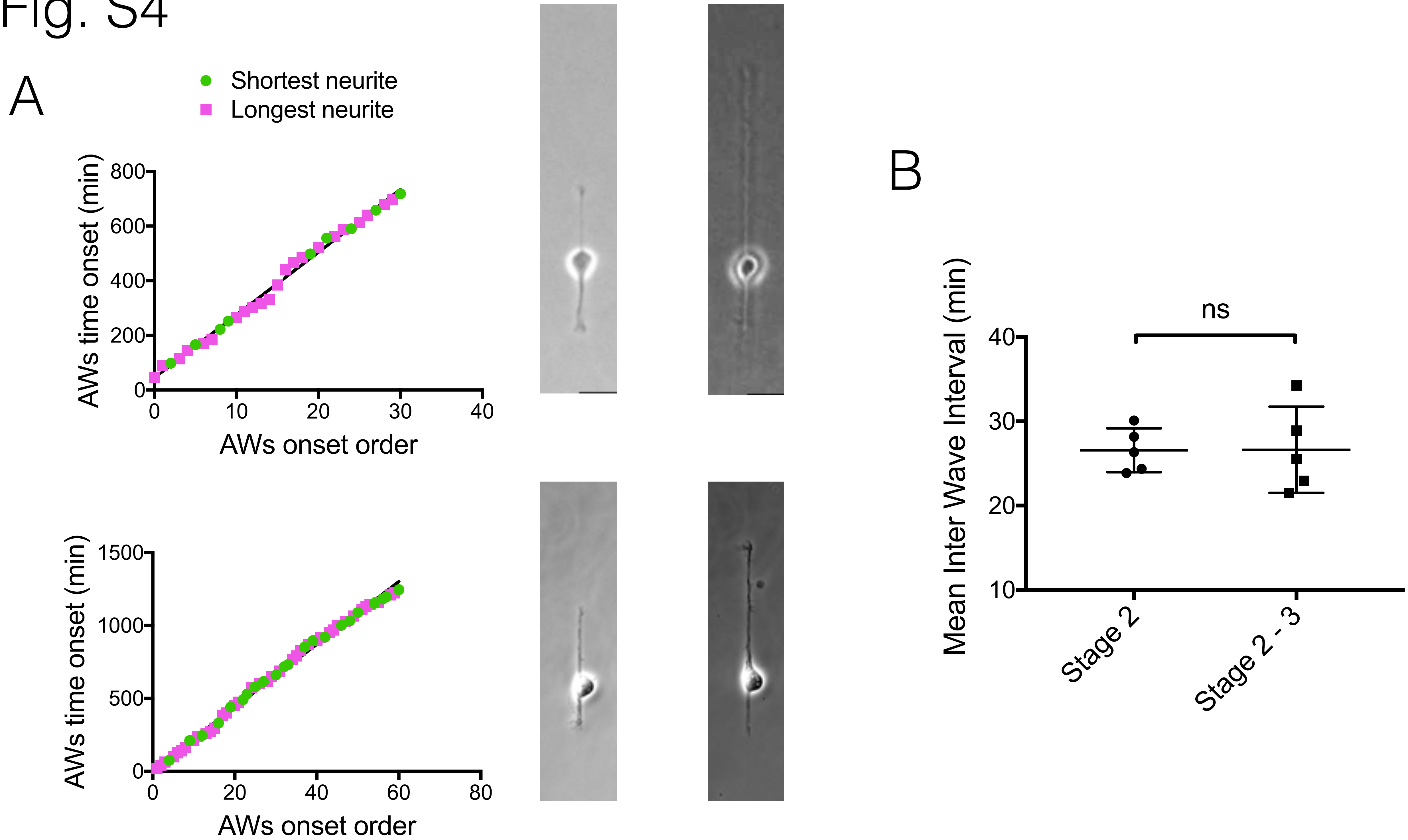

Fig. S4 - Evolution of the inter waves intervals over neuronal growth. A- The graphs display the times where successive actin waves are observed (the slopes of the rather linear variations displayed here give the mean inter wave intervals of each cell). Purple symbols: actin wave time onset on the longest neurite. Green symbols: actin wave time onset on the shortest neurite. The images of the cells at the start and the end of the time-lapse experiments corresponding to the graphs show an evolution from a rather symmetric to a clearly asymmetric morphology (transition from stage 2 to stage 3 neurons). Nevertheless, the regularity of the time onset of actin waves at the soma level is not affected, showing that the inter wave interval is not modified through growth and morphological polarization. B- Mean inter wave intervals of stage 2 and stage 2-3 neurons. Five cells for each conditions, totalizing 207 (stage 2 neurons) and 203 (stage 2-3 neurons, i.e. symmetric neurons becoming asymmetric during observation) IWIs. ns: p=0.841 (two-tailed Mann-Whitney U test).

Fig. S5

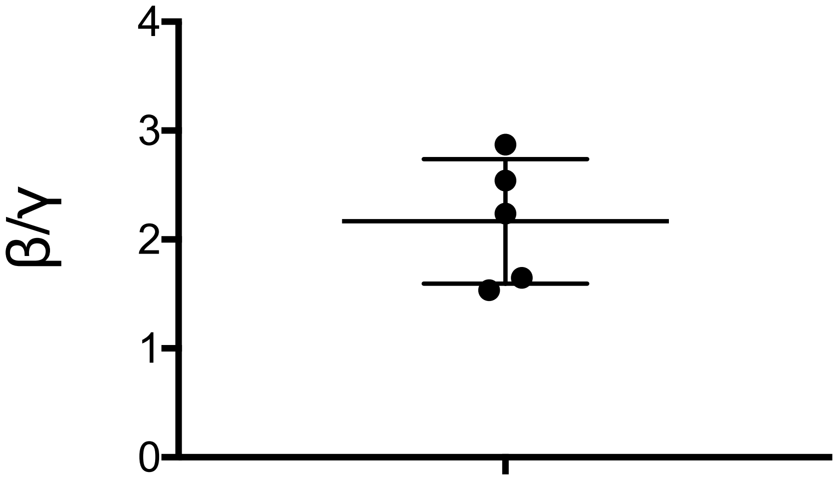

Fig. S5 - Ratio of the coefficients β and γ expressing the accelerated growth of the nascent axon (β > 1) and the reduced growth of the competing dendrite (γ <1), respectively compared to the single neurite growth rate before polarization. 5 cells.

Fig. S6

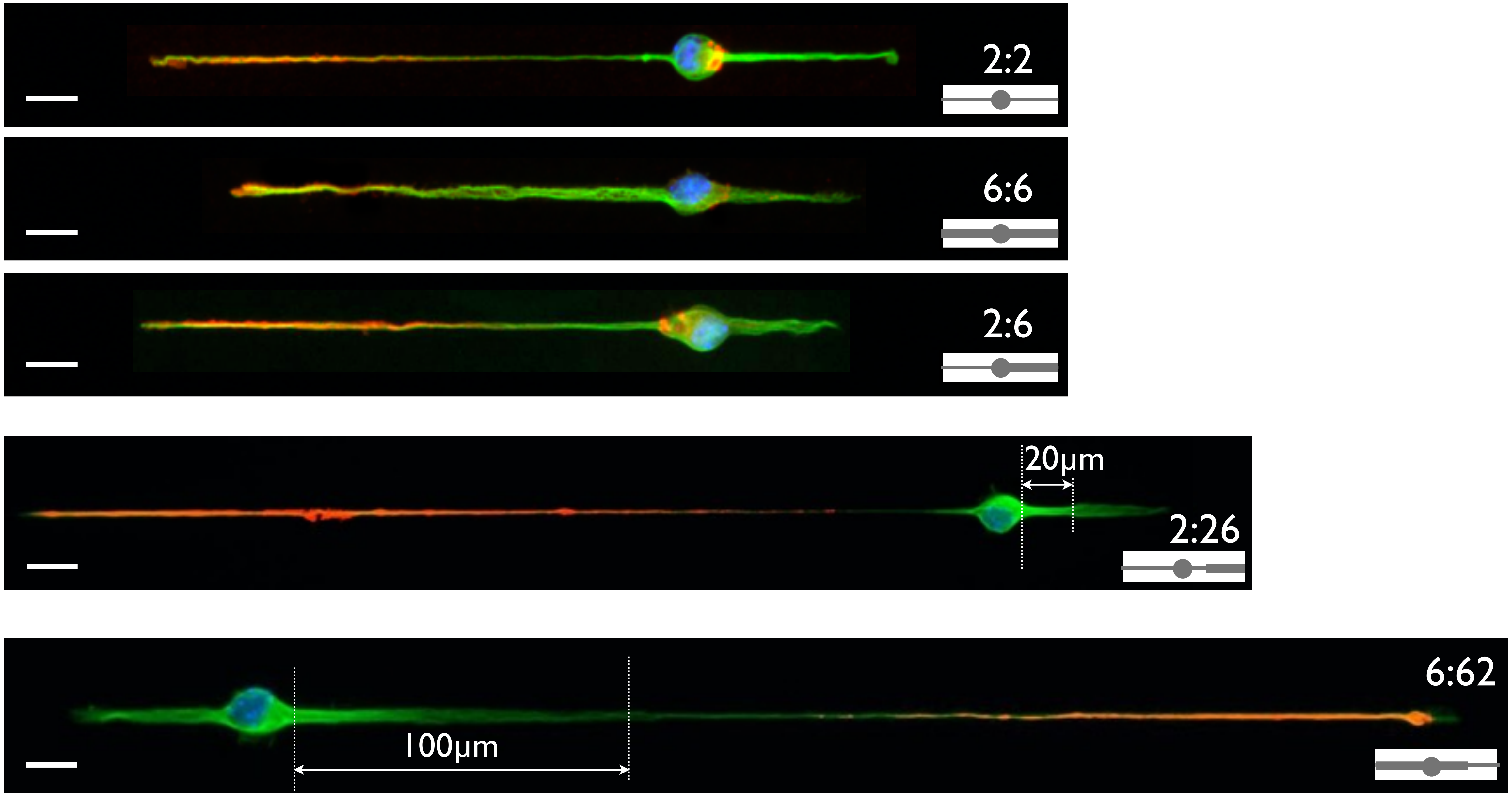

Fig. S6 - Images of polarized neurons on different patterns (*i.e.* 2:2, 6:6, 2:6, 2:26 and 6:62 patterns) showing a few examples of the cell morphologies produced according to the patterns shown in the insets. For the 2:26 and 6:62 patterns, the dashed lines indicate the transition between two stripe widths, and numbers in μm the length of the proximal stump.
Microtubules (YL1/2, green), nucleus (Hoechst, blue) and axon (Tau-1, red).
Scale bars : 15μm.

Fig. S7

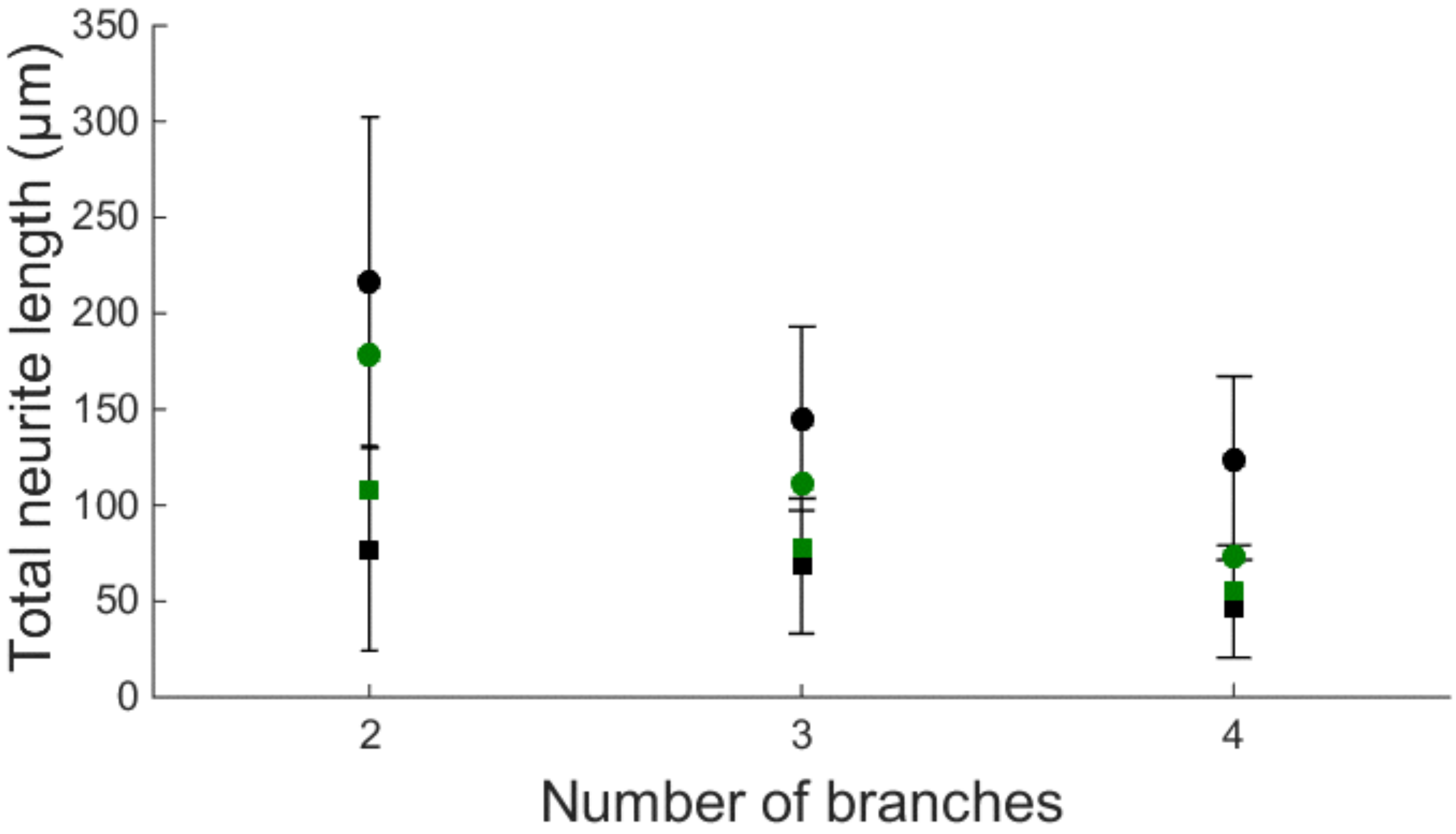

Fig. S7 - Neurite lengths on the 2, 3 and 4 branches patterns. The black symbols denote the experimental data: solid circles represent the measured longest neurite, and squares the mean of the shortest neurites. Mean values (longest, shortest): (216.7μm, 77.0 μm) for neurons with 2 branches (278 cells), (145.2μm, 68.3μm) for neurons with 3 branches (37 cells), and (123.2μm, 46.1μm) for neurons with 4 branches (118 cells). Green symbols (circles: longest neurite, squares: mean of the shortest neurites) represent the results from our model.
